# Maintenance of intervention effects: long-term outcomes for participants in a group talk-therapy trial in the Democratic Republic of Congo

**DOI:** 10.1017/gmh.2022.39

**Published:** 2022-07-26

**Authors:** Judith K. Bass, Sarah M. Murray, Daniel P. Lakin, Debra Kaysen, Jeannie Annan, Amani Matabaro, Paul A. Bolton

**Affiliations:** 1 Department of Mental Health, Johns Hopkins Bloomberg School of Public Health, Baltimore, MD, USA; 2 Department of Psychiatry and Behavioral Sciences, Stanford University, Stanford, CA, USA; 3 The International Rescue Committee, New York, NY, USA; 4 Action Kivu, Bukavu, Democratic Republic of Congo

**Keywords:** Gender-based violence survivors, long-term follow up, trauma intervention

## Abstract

**Background:**

Despite the growth of psychotherapy trials in low- and middle-income countries, there have been limited follow-up studies of more than 2 years. This study follows up female sexual violence survivors approximately 6 years after completing a 12-session group cognitive processing therapy (CPT) program in the eastern Democratic Republic of Congo.

**Methods:**

Baseline trial data were collected in December 2010 from 134 women in 7 study villages randomly allocated to CPT. Study women were over 18 years, reported personally experiencing or witnessing sexual violence, and reported elevated depression, anxiety and/or posttraumatic stress symptoms. Women were followed up (1) post-treatment (6-months after baseline); (2) 6 months later; (3) 12 months later; and (4) in March 2017 (6.3 years after baseline). At the long-term follow-up, 103 women (77%) in 6 of 7 CPT villages were re-assessed; one village was not visited due to ongoing insecurity.

**Results:**

We found strong continued intervention effects; nearly all women maintained treatment impacts over the first two years; at long-term follow-up, approximately half continued to maintain low symptom scores. Relapse rates for probable PTSD and probable depression and anxiety were 20%.

**Conclusions:**

This study extends prior research to show that treatment impacts can be maintained for several years despite experiences of ongoing trauma. The women described continuing to meet with the women in their therapy group and using the skills they learned in the psychotherapy, providing evidence of the potential for these programs to provide valuable social supports and skills that people use as they continue to face adversity.

## Introduction

Mental health problems such as depression, anxiety and posttraumatic stress are common globally and elevated in areas of high poverty and instability (Lund *et al*., 2010; Banks *et al*., 2018). The last two decades have a growth in treatment trials in low- and middle-income countries designed to determine what kind of interventions are effective in reducing the burden of these problems (Barry *et al*., 2013; Chibanda *et al*., 2015; Purgato *et al*., 2018) and how these interventions can be implemented in contexts with few formal mental health professionals or services (Joshi *et al*., 2014; Munodawafa *et al*., 2018). Many studies have found that intervention effects wane after 3–6 months (Purgato *et al*., 2018); and for those that have found continued effects (Bass *et al*., 2006; Maselko *et al*., 2015), there is a lack of data on the extent to which impacts are sustained several years later.

Data from high-income country studies suggest that impacts of cognitive-behavioral therapies may persist beyond the typical 6–12 month study follow-up periods (DiMauro *et al*., 2013). A book by Dugas and Robichaud (2007) on the effects of cognitive-behavioral treatment for generalized anxiety disorder (GAD) found that more than half of intervention participants remained asymptomatic from two to 14 years. For depression, a meta-analytic review found that approximately 60% of individuals who received psychotherapy remained remitted more than two-years following treatment (Steinert *et al*., 2015). Among sexual assault survivors, follow-up research by Resick and colleagues found that cognitive processing therapy (CPT) treatment effects were maintained for depression and PTSD symptoms at three, six, and nine months post treatment for a group of women who had experienced sexual trauma (Resick *et al*., 2002) and then again at ‘long term follow-up’, ranging from 5 to 10 years after treatment (Resick *et al*., 2012; Wachen *et al*., 2014).

Multiple theories have been postulated for how treatments might continue to confer benefits long after the formal treatment period is complete. Cognitive theory posits that cognitive therapy teaches skills in how to identify and change maladaptive cognitions, and that the change in processing this information is what leads to the maintenance of symptom change (Beck, 2005). In CPT specifically, changes in trauma-related beliefs predict symptom improvement during treatment (Sobel *et al*., 2009) and reductions in maladaptive trauma-related beliefs predict lower PTSD and depressive symptoms 5–10 years after treatment (Iverson *et al*., 2015). At the same time, context matters and factors such as lower socioeconomic status predict poorer long-term functioning following treatment (Wachen *et al*., 2014).

To our knowledge, there have been no long-term (> 2 years) follow-up of psychotherapy studies for common mental health problems in a low-income country, particularly one where ongoing instability and conflict is common with populations continually being exposed to a myriad of traumas. This study looks at mental health outcome data collected approximately 6 years after intervention completion among women who participated in a 12-session group CPT program in the eastern Democratic Republic of Congo. The research questions are: (1) what proportion of women maintained the symptom reductions observed 1-year posttreatment and (2) what client- or treatment-level variables were associated with continued treatment impact.

## Methods

The parent study (Bass *et al*., 2013) included 405 adult women who had reported personally experiencing sexual violence and presented with moderate to severe symptoms of depression, anxiety and/or PTSD and impaired functioning; these women lived in 15 study villages that were randomly allocated to the CPT (*N* = 7 villages) or individual support conditions (*n* = 8 villages). Baseline assessment was completed in December 2010 and the intervention period was from April through July 2011 with CPT provided by local psychosocial workers trained and supervised by US-based CPT trainers. Primary study outcomes were reduction in symptoms of depression, anxiety and posttraumatic stress and improvement in functional impairment. Treatment impacts at 6-month post treatment were previously reported (Bass *et al*., 2013); briefly CPT, as compared with individual support alone, was effective in reducing PTSD symptoms (effect size 1.3) and combined depression and anxiety symptoms (effect size 1.6) and improving functioning (effect size 1.2) in this sample of female survivors of sexual violence. Following the 6-month follow-up, women in the CPT villages were invited to participate in a year-long social-economic program (Village Savings and Loans Associations; VSLA) and were re-assessed a fourth time after that program was completed; ninety-seven women participated in the VSLA program.

**Fig. 1.**
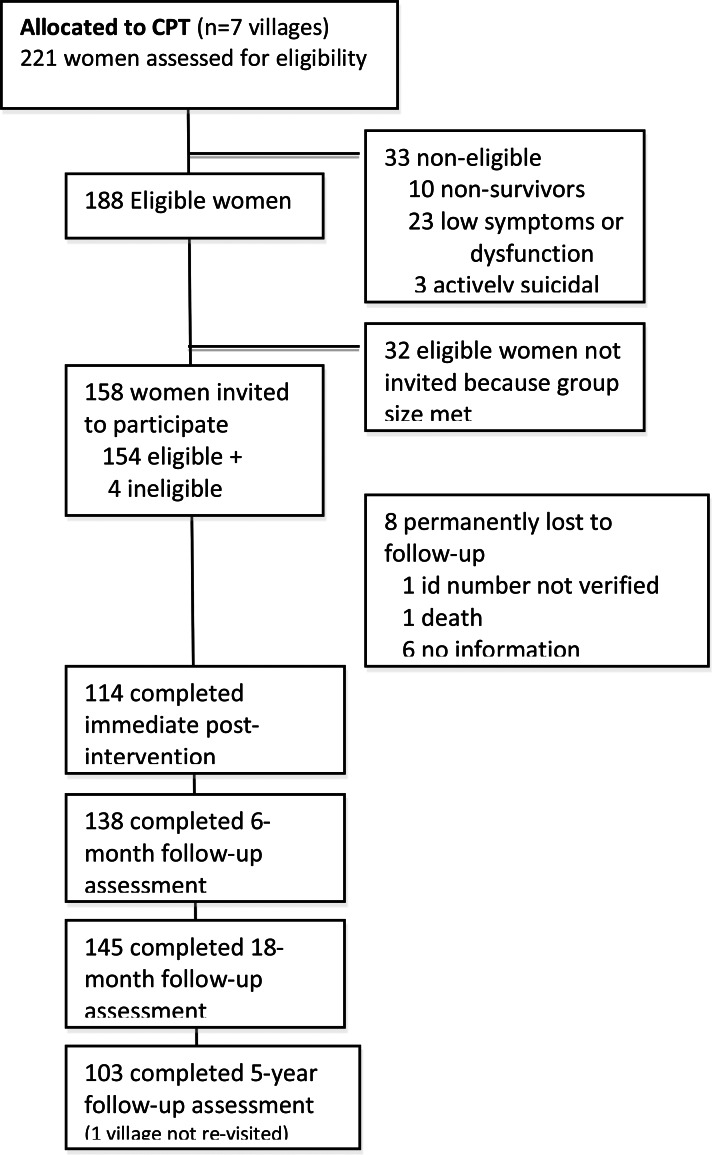
Participant flow chart.

For the current follow-up, which occurred in March 2017 approximately 6.3 years after baseline, women in 6 of 7 CPT villages were approached; one village was not visited due to ongoing security issues and the individual support villages were not visited as potential comparators as their psychosocial workers had subsequently been trained in CPT and provided the treatment to those still needing services. Figure 1 shows the flow of CPT participants for each of the five data collection time points from the study baseline through the long-term follow-up (see Table 1 for demographics at baseline and long-term follow-up).

**Table 1.** Characteristics of CPT trial participants at baseline and long-term follow-up

	Sample at baseline (*N* = 134)	Sample at long term follow-up (*N* = 103)
*Demographics*		
Age in years	38.60 (13.3)	43.6 (12.7)
Number of people living in home	7.70 (3.1)	8.6 (3.6)
Number of children responsible for	4.24 (2.7)	4.1 (2.2)
Currently pregnant	12 (9.0%)	10 (9.7%)
Marital status		
Single	12 (9%)	10 (9.9%)
Married	85 (63%)	57 (56.4%)
Divorced/Separated	16 (12%)	7 (6.9%)
Widowed	21 (16%)	27 (26.7%)
Living with husband (if married)	69 (81%)	49 (86.0%)
*Prior traumas*^a^		
Number types of trauma experienced	3.87 (1.1)	1.0 (1.5)
Number types of trauma witnessed	5.25 (1.1)	2.8 (2.1)
Experienced sexual violence, n (%)	126 (94.0)	16 (16.8%)
Witnessed sexual violence, n (%)	111 (82.8)	43 (45.3%)

a At baseline prior trauma was assessed over lifetime; at long-term follow-up, prior trauma was assessed for the previous 6 months.

### Measures

To assess PTSD symptoms, we utilized the 16-item Harvard Trauma Questionnaire (HTQ; Mollica *et al*., 1992) symptom section and to assess depression and anxiety symptoms we used the Hopkins Symptom Checklist (HSCL-25; Hesbacher *et al*., 1980; Winokur *et al*., 1984) which includes 15 depression symptoms and 10 anxiety symptoms. The mental health measures were adapted and pilot-tested in each of the three study language groups (Mashi, Kifuliro and Swahili). Both the HSCL-25 and the HTQ have been used internationally with sexual-violence survivors and conflict-affected samples generally (Halepota and Wasif, 2001; Kleijn *et al*., 2001; Ventevogel *et al*., 2007; Rasmussen *et al*., 2015; Wind *et al*., 2017). Participants rated the frequency of each symptom in the prior 4 weeks on a four-point Likert scale (with 0 denoting not at all, 1 a little bit, 2 a moderate amount, and 3 a lot). Average per-person scores were generated for each measure, with scores ranging from 0 to 3; higher scores indicate greater severity. An average score of 1.75 or higher on either measure was considered to be predictive of clinically significant symptoms on the basis of data from other conflict-affected populations (Mollica *et al*., 1987, 1992). Both measures were completed at all assessment points, yielding five data points.

Other self-report measures administered at the long-term follow-up assessment included basic demographic characteristics, a locally-developed measure of daily functioning, an index of past 6-month trauma experiences, continued use of each CPT skill including examples of when the skills were used, and continued meetings with CPT group participants. The daily functioning measure consists of 20 tasks of daily living that focus on activities related to caring for one's self, one's family, and one's community. Participants rated the amount of difficulty they had completing each task in the prior 4 weeks on a five-point Likert scale (with 0 denoting no difficulty at all, 1 a little bit of difficulty, 2 a moderate amount of difficulty, 3 a lot of difficulty, and 4 unable to complete the task). Average per-person scores were generated, with scores ranging from 0 to 4; higher scores indicate greater functional impairment.

### Intervention

CPT is a protocol-based therapy with strong evidence for reducing the burden of depression, anxiety, and PTSD symptoms in sexual-violence survivors. For this study, CPT was provided in a group format with 6–8 women per group. We used the cognitive-only version of the treatment (i.e. without a trauma narrative) because its efficacy is similar to that of the full version of the therapy (Resick *et al*., 2008), while providing greater ease of administration in groups. The treatment included 1 individual session (1 hour) and 11 sessions with six to eight women per group (2 hours each). The therapy was adapted to meet the needs of a predominantly illiterate population who were at risk for exposure to ongoing violence (Bolton *et al*., 2014).

The psychosocial assistants (PSA) who provided therapy were village-based Congolese women who had been providing general psychosocial care to sexual violence for at least 1 year prior to the trial; none had prior mental health training. The PSAs received 2 weeks of in-person training with CPT trainers from the United States in the use of a manual that was adapted to the local context and educational level of the PSAs (most had completed secondary education) and translated locally. Direct supervision was provided by Congolese psychosocial supervisors who were employees of The International Rescue Committee (IRC), the partner research and implementing organization, through weekly telephone or in-person meetings. A bilingual clinical social worker trained in the U.S. supervised the psychosocial supervisors and communicated with the U.S. trainers weekly to monitor quality and fidelity. Each PSA concurrently led three CPT groups.

### Data analysis

Descriptive statistics were calculated for demographic and symptom variables at baseline for the full baseline sample from the six targeted villages randomized to CPT and separately for women who completed the long-term follow-up. Characteristics were compared for women in these villages who did and did not participate in the long-term follow-up using student's *t* tests and Fisher's exact tests due to small cell sizes. We also calculated descriptive statistics for women's responses to questions about the frequency of meeting with CPT groups and the types of activities they engaged in related to use of CPT skills.

To assess differences in mental health and functional impairment outcomes at long term follow-up as compared to pre-CPT, baseline mean symptom and function scores were compared to mean scores at each subsequent time point (post-treatment, 6-months post-treatment, 18-months post-treatment, and at the long-term follow-up approximately 6 years-post-treatment) using paired *t* tests for continuous mean symptoms scores and McNemar's test for binary probable diagnosis using all available data from women who participated in the long-term follow-up.

For the subsample of women (*n* = 76) who met our baseline criteria for probable clinically significant symptoms (a score of 1.75 or higher on either measure), we explored remission and recovery over the follow-up period. Remission was defined as dropping below the cut-off score at one of the follow-up assessments but then moving back above the cut-off at a later follow up. Recovery was defined as dropping below the cut-off and remaining below the cut-off at all follow-up assessments.

To assess the extent to which initial symptom changes after participation in CPT were maintained in the long-term for all study women, we categorized women based on symptom score changes from immediate post-treatment or six-months post-treatment (whichever was lower) to final assessment. We categorized women into those who: (1) maintained reductions: whose symptom score changed by only a small amount (⩽0.5 points); (2) women who partially maintained reductions: whose symptom score increased by more than 0.5 but less than 1.0 points; or (3) women who did not maintain their reductions: whose symptom scores increased by 1.0 points or more. The possible symptom score range for the mental health measures is 0 to 3.0 points (0 = not experiencing the symptom at all; 1 = some of the time; 2 = a moderate amount of time; 3 = all the time); an increase in 1.0 suggests a full category change.

We used multinomial logistic regression to assess whether the following characteristics of women recorded at study baseline were associated with symptom maintenance by comparing women who maintained symptom reductions (group 1) with women who partially maintained reductions (group 2) and women who did not maintain reductions (group 3): age, marital and pregnancy status, whether they had lived in the area for 10 years or more, number of people living in the home, number of children women were responsible for, total number of types of traumas witnessed, total number of types of traumas experienced, and functional impairment, as well as depression, trauma, and total symptoms. These are the same predictors that were explored in the original trial paper (Bass *et al*., 2013). In addition, we assessed whether the number of CPT skills a woman still used, participation in a VSLA group, number of types of trauma experienced or witnessed in the past six months, and frequency with which a woman had met with her CPT group in the past six months as reported at this follow-up was associated with the maintenance classifications.

The Institutional Review Boards (IRB) of the Johns Hopkins University and the Kinshasa School of Public Health approved the original trial protocol; the Johns Hopkins University IRB approved the amendment for the current follow-up. The sample of women who participated in the CPT treatment groups in the original trial was contacted by their local psychosocial staff and personally invited to participate in this follow-up interview; consent was obtained by study interviewers for those women who agreed to participate.

## Results

Of the 134 women from the treatment arm in the 6 follow-up villages of the original trial, 16 (12%) were unable to be located, had passed away, or were no longer living in the study area. In addition, 15 women (11%) declined or were unable to participate. Thus, 103 (77%) participated in this follow-up study; 99 (96%) of these women participated in at least 2 previous follow-up assessments. Table 1 presents the demographic characteristics of the full treatment sample and those who participated in the long-term follow-up at baseline and the follow-ups characteristics at the time of the 7-year follow-up. At this follow-up, the women continued to live in homes with multiple people and were responsible for, on average, four children. The percent of women married went down (from 65% to 56%) while the percent of widowed women went up (from 16% to 27%). At baseline, the study women reported a lifetime history of having experienced and witnessed a range of different types of traumas. Nearly 17% of the women reported experiencing sexual violence in the 6-months before the final assessment was completed, with 45% reported having witnessed sexual violence.

### Symptom severity and functional impairment outcomes at each follow-up

**Fig. 2.**
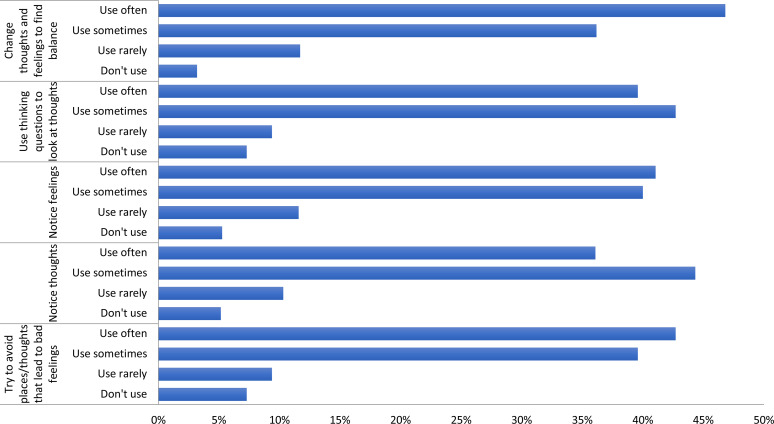
Frequency of specific CPT skill use.

The trajectory of symptom changes for the 103 women in this follow-up study did not differ across the mental health and function outcomes (Fig. 2). Looking at each time point (Table 2) the average baseline scores were close to 2.0, indicating that on average, the women experienced nearly all of the symptoms ‘a moderate amount’ of the time. For the first 18 months after the treatment was complete, the women maintained average symptom scale scores below 1.0, indicating that on average the women experienced all of the symptoms less than ‘a little bit’ of the time. At the final follow-up, the average scores had increased, but remained clinically and significantly less than initial baseline scores. Similar patterns were seen for the outcome of functioning, with impairment remaining down from baseline levels even at the final follow-up.

**Table 2.** Symptom and function scores across follow-up assessments for the *N* = 103 sample

Variable	Mean (s.d.)^a^	Mean difference from baseline (se)^b^	*p* value^c^
HSCL-25 score for depression/ anxiety			
Baseline	2.06 (0.5)		
End of treatment	0.80 (0.7)	−1.26 (0.09)	<0.0001
6 months after end of treatment	0.66 (0.6)	−1.40 (0.08)	<0.0001
18 months after end of treatment^d^	0.78 (0.7)	−1.27 (0.09)	<0.0001
Long-term follow-up	1.21 (0.7)	−0.85 (0.09)	<0.0001
HTQ score for PTSD			
Baseline	1.96 (0.5)		
End of treatment	0.74 (0.6)	−1.22 (0.09)	<0.0001
6 months after end of treatment	0.63 (0.6)	−1.33 (0.09)	<0.0001
18 months after end of treatment^d^	0.71 (0.7)	−1.25 (0.10)	<0.0001
Long-term follow-up	1.09 (0.7)	−0.87 (0.09)	<0.0001
Functional impairment score			
Baseline	1.67 (0.7)		
End of treatment	0.78 (0.7)	−0.94 (0.12)	<0.0001
6 months after end of treatment	0.72 (0.6)	−0.93 (0.09)	<0.0001
18 months after end of treatment^d^	0.96 (0.8)	−0.73 (0.11)	<0.0001
Long-term follow-up	1.21 (0.7)	−0.47 (0.09)	<0.0001

a Mean and s.d. on the basis of available data at each time point. PTSD denotes post-traumatic stress disorder.

b We report the paired test for mean difference in score at each follow-up to baseline.

c Significance of difference in mean score from baseline.

d This follow-up assessment occurred after the women had participated in the VSLA program.

### Diagnosis, remission and recovery

At baseline, 74% (*n* = 76) of the CPT participating women met criteria for a probable diagnosis of clinically significant depression and anxiety at baseline. 9 (12%) of these women did not move into remission at the post-intervention assessment and of these, 6 remained above the cut-off at the final long-term assessments. Among the 67 women who remitted at the post-intervention assessment, 53 (79%) would be classified as recovered at the long-term follow-up assessment and 14 (21%) as having relapsed. For the posttraumatic stress symptoms, 61% (*n* = 63) of the CPT participating women met criteria for a probable diagnosis at baseline. Of these, 4 (6%) did not move into remission at the post-intervention assessment and 2 of these women remained above the cut-off at the long-term follow-up. For the 59 women who remitted at the post-intervention assessment, 47 (80%) would be classified as recovered at the long-term follow-up assessment and 12 (20%) as having relapsed.

Table 3 presents results from the analysis to explore the maintenance of total symptom score reductions during period between the last two assessment periods (18 months after treatment to long-term follow-up). Maintenance was defined as having scores increase or decrease by less than 0.5 points. Analyses to explore predictors of symptom maintenance or worsening did not identify any significant demographic or clinical factors.

**Table 3.** Maintenance of intervention effects at final follow-up (*n* = 101)^a^

Reductions in symptoms	Maintained, *n* (%)^b^	51 (50.5)
	Partially maintained, *n* (%)^c^	24 (23.8)
	Not maintained, *n* (%)^d^	26 (25.7)

a 2 women were missing immediate post and 6-month follow-up scores.

b Women whose scores further reduced or increased by less than or equal to 0.5 points are considered maintainers.

c Women whose scores increased by more than 0.5 but less than 1.0 points.

d Women whose scores increased by 1.0 points or more.

### CPT program outcomes

At the long-term follow-up assessment, 90 (87%) women reported that they had continued to meet with the other women from their CPT groups, with 16 (18%) indicating they had met once or twice in the past 6 months, 17 (19%) indicating they had met about once a month, 29 (32%) reported meeting once per week and 24 (27%) reported meeting more than once per week. The activities they reported engaging in with the women from their group ranged from discussing their problems and feelings, socializing, and doing microfinance or other income generation activities. When asked directly, nearly all of them (97%) reported they used CPT skills to help one another.

When asked about their ongoing use of specific skills learned during the CPT program, more than 75% of them women reported sometimes or often using each of the 5 CPT-specific skills.

## Discussion

We found strong continued intervention effects in this long-term follow-up of 103 women who participated in a group psychological treatment program in rural DRC. Nearly all of the women maintained the initial treatment impacts during the first 18 months after treatment was complete, and after more than 5 years from the end of treatment, approximately half continue to maintain their relatively low symptom scores. This despite ongoing regional instability and women's continued exposure to traumatic events including sexual violence. Nearly 1 in 5 women reported experiencing sexual violence in the 6-months prior to this final assessment; this is approximately twice the number of women who reported sexual violence in the long-term follow up of rape survivors participating in CPT in the U.S. (Resick *et al*., 2012). This is also better than would be expected in the absence of an available treatment; in a systematic review of longitudinal studies of populations with PTSD, the two studies that included patients who had experienced rape, assault and other types of violence reported recovery rates of 18% and 28% after 5 years (Steinert *et al*., 2015). These results were also robust for disorder designation, as we saw similar patterns for the PTSD symptom measure (HTQ) and for the depression/anxiety symptom measure (HSCL-25) and the conclusions did not change when analyses were done looking at the scores dimensionally or by probable diagnosis categorization.

Relapse rates for both the probable PTSD diagnoses and for the depression and anxiety combined diagnosis were around 20%; this is similar to what was found in the long-term follow up with female rape survivors in the U.S. (Resick *et al*., 2012). A similar percent was identified as having significant symptom worsening between the 18-month follow-up and the long-term follow-up assessment. In our exploratory analyses of potential predictors of relapse and symptom worsening, we did not identify any significant demographic or trauma-related factors. Continuing to meet with the other women from their groups was common and participation variables also did not predict relapse or symptom worsening.

Despite a formal CPT program no longer being formally implemented by the providers in their communities due to lack of financial support for program implementation, many women reported that they continued to meet with the women from their group and reported continued use of the CPT skills. In some clinical settings in the US providers may have the option to provide booster CPT sessions if patients are demonstrating worsening symptoms or if they are faced with a new traumatic event (Resick *et al*., 2016). It is possible that the ongoing availability of the APS providers allowed women to get ongoing CPT-related support from them despite this not being formally part of the program. As the women spoke about using the CPT skills with the other women from their original group, it is also possible that these ongoing group sessions provided informal boosters from their peers. Women who participated in the CPT program as part of the trial were also invited to participate in a social-economic program (VSLA) that was implemented between the 6- and 18-month follow-up assessments and at the final follow-up, women did report doing microfinance activities with their groups.

This speaks to the potential mechanisms by which the women continued to benefit from the original CPT program. The CPT treatment program provided the women with cognitive skills that they reported continuing to use to deal with the violence and ongoing adversity in their lives. This is particularly relevant as this region of DRC continues to experience instability and conflict and sexual violence continues to be prevalent (Guterres, 2019). This supports the theory that teaching cognitive and emotional skills, and the use of cognitive restructuring, may indeed be a mechanism for the maintenance of symptom gains. In CPT, group members are taught to use the cognitive skills to help challenge each other's maladaptive cognitions, which may also help maintain treatment effects.

Another potential mechanism is the group-based delivery of the psychotherapy, which created access to a trusted group of peers who have similar skills to provide support and ongoing advice. Previous research with this sample showed that the group CPT participants experienced more structural social capital and emotional support compared with participants in the individual support condition (Hall *et al*., 2014), which may have been directly related to the support from the other group members. A long-term follow up of group-based interpersonal psychotherapy (IPT) found that the groups continued to meet several years later (Lewandowski *et al*., 2016), so this finding of continued group meetings may be an important factor to explore in future group-based treatment studies.

We did not find participant-level characteristics (demographics, recent trauma history, initial baseline scores) that differentiated which women were able to maintain the treatment impacts. Some potentially important factors, including cumulative trauma exposure and other life events that may have occurred in the intervening period were not captured in our assessments. Consistent with past research on predictors of poorer long-term outcomes in CPT, it is possible that the economic hardships faced by women in these communities may also be a factor in the higher rate of relapse in this sample, as compared to US samples (Wachen *et al*., 2014).

There are several limitations that must be noted, including the lack of an available comparison group as the women in the original control villages received CPT after the trial was complete. In addition, there are unmeasured predictors that may be important that were not collected as part of this study including cumulative trauma exposure, change in trauma-related cognitions (Iverson *et al*., 2015), which might have provided further detail on potential mechanisms of treatment maintenance. We also did not look at the symptom level to see whether there were specific symptoms that were more treatment resistant or more likely to re-occur than others (Larsen *et al*., 2019). Individual symptoms that are more prone to re-occur or are treatment resistant such as sleep, detachment, fatigue, or guilt might provide directions for refining interventions. Finally, we have limited data on what other programs may have been implemented in the study communities over the 5-year follow-up period; anecdotally the women did not report any major NGO or government initiatives, nor any new mental health programs in their communities.

The original trial was the first to implement CPT with lay providers for sexual violence survivors in a context of ongoing conflict and sexual violence; the results indicated strong impacts of CPT participation on reducing the burden of depression, anxiety and PTSD symptoms and improving daily functioning. This long-term follow-up study shows that after 5 years from the final post-treatment follow-up, most CPT participants continue to meet with their original CPT groups, continue to use the cognitive skills that are fundamental to the CPT model, and continue to live with relatively low symptom burdens despite the presence of ongoing violence and conflict. This despite the CPT providers not having continued formal support to deliver this intervention, which is a challenge of many programs that begin as studies but are not embedded within existing systems of care. This study provides evidence that psychotherapies can act not only as treatments for reducing the burden of mental illness but can also act as prevention programs that can provide valuable skills that people can use as they continue to face adversity. This study also provides an argument for considering the long-term feasibility of implementing these types of programs from the initiation of the studies, including identifying systems to maintain the employment of trained providers.
